# Explainable recommendation: when design meets trust calibration

**DOI:** 10.1007/s11280-021-00916-0

**Published:** 2021-08-02

**Authors:** Mohammad Naiseh, Dena Al-Thani, Nan Jiang, Raian Ali

**Affiliations:** 1 Faculty of Science and Technology, Bournemouth University, Fern Barrow, Poole, BH12 5BB UK; 2 College of Science and Engineering, Hamad Bin Khalifa University, Doha, Qatar

**Keywords:** Explainable AI, Trust, Trust Calibration, User Centric AI

## Abstract

Human-AI collaborative decision-making tools are being increasingly applied in critical domains such as healthcare. However, these tools are often seen as closed and intransparent for human decision-makers. An essential requirement for their success is the ability to provide explanations about themselves that are understandable and meaningful to the users. While explanations generally have positive connotations, studies showed that the assumption behind users interacting and engaging with these explanations could introduce trust calibration errors such as facilitating irrational or less thoughtful agreement or disagreement with the AI recommendation. In this paper, we explore how to help trust calibration through explanation interaction design. Our research method included two main phases. We first conducted a think-aloud study with 16 participants aiming to reveal main trust calibration errors concerning explainability in AI-Human collaborative decision-making tools. Then, we conducted two co-design sessions with eight participants to identify design principles and techniques for explanations that help trust calibration. As a conclusion of our research, we provide five design principles: Design for engagement, challenging habitual actions, attention guidance, friction and support training and learning. Our findings are meant to pave the way towards a more integrated framework for designing explanations with trust calibration as a primary goal.

## Introduction

Machine learning (ML) models can be designed to appear as a black box where end-users cannot or are not required to understand “Why” the system makes a particular recommendation [38, 43, 93]. The motivation for adopting the black-box modality is mainly due to preserving the confidentiality of the algorithm and its competitiveness, e.g., when they yield high accuracy results. However, this introduces a lack of transparency which can weaken the users’ ability to calibrate trust in the Human-AI collaborative decision-making environments. This means the human decision-maker is less able to judge when to follow a system output or reject it [33]. These challenges have motivated scholars and researchers in eXplainable Artificial Intelligence (XAI) to develop explainable algorithms and models to increase the transparency and interpretability of ML models.

A significant work in XAI has focused on introducing novel explanation techniques with a focus on two primary methods [2, 91]. The first explanation type is model-specific, in which ML models are designed to be inherently explainable and transparent. This is often seen in rule-based algorithms. The second is model-agnostic which can be used to interpret any machine learning model by extracting knowledge from the model and its predictions. This is seen in LIME which approximates a locally interpretable model, e.g. linear model, for a black-box model to explain it [67]. Despite the growing body of knowledge on XAI [2, 55], little focus has been given to studying how human decision-makers utilise explanations during a Human-AI collaborative decision-making task. In a recent survey in XAI, Adadi and Berrada [2] found that XAI research has focused on developing explanation models with high fidelity rather than understanding how users would interact and interpret these explanations. Another research [49] showed that current explainable models are designed to be used by ML engineers for debugging purposes where end-users are not the main target of these explanations. These findings indicate that operationalising recent advances in XAI literature in real-world scenarios to meet user experience is still a new area to discover. Also, user experience (UX) in XAI, specifically Human-AI collaborative decision-making, may require more attention than the explanation ease of use [47]. UX in such context means that the system shall ensure conveying the message without requiring a high cognitive load and introduce minimal possible interruption to the Human-AI task. For instance, distraction in a typical system is to avoid, but it might not be the case for XAI as a distraction may be needed to nudge people on looking at an explanation.

Recently, trust calibration became an essential metric for evaluating collaborative Human-AI decision-making tools [7, 16, 96]. The role of XAI in trust calibration refers to the extent to which explanations and their communication method are helping to form a correct mental model of the AI-based tool; thus, the human decision-maker is more informed on whether to trust or distrust the AI recommendations. The research argued that when mistakes happen, e.g., over-trusting an incorrect recommendation or under-trusting a correct recommendation, it can lead to critical consequences. Several studies have investigated the relationship between trust calibration and explanations [7, 16, 96]. They found that communicating explanations, on average, is not improving trust calibration, i.e., users still end-up in situations where they over-trust or under-trust AI-based recommendation. Indeed, several studies discussed reasons and situations where explanations did not improve trust calibration, e.g., explanations were perceived as an information overload by the humans’ decision-makers [55]. However, the research still lacks structured and specific studies that propose design solutions to enhance the role of explanation in trust calibration. To acquire such knowledge, we follow a user-centric approach as it enables obtaining empirical evidence for designing explanations for specific goals, domain and population [1].

In this paper, we devise XAI design techniques and principles for XAI interfaces to enhance the role of explanations in calibrating users’ trust. We used screening prescription as a Human-AI collaborative decision-making task where the human medical practitioner uses the AI to check whether the prescription can be approved for a given patient. Such a task reflects an everyday Human-AI collaborative decision-making task where trust calibration errors are possible. We follow a multi-stage qualitative research method, including think-aloud protocol and co-design sessions with medical practitioners. Our results shed light on the nuances of the lived experiences of XAI users and how the design can help their trust calibration. The collected data included three main data sets: (i) researcher observations of participants interacting with AI-based explanations, (iii) transcribed audio files of the multi-stage qualitative study, and (iii) participants sketches of the AI-based explanations. As a result, and to mitigate the observed trust calibration errors in participants’ experience with XAI, we propose five design principles that could enhance the role of explanations in trust calibration: design for engagment, challenging habitual actions, attention guidance, friction, as well as support training and learning.

The paper is structured as follows. In Sect. 2, we provide a theoretical background and related work. In Sect. 3, we describe the research method followed in our study, including the sample, material and instruments used, and our analysis. In Sect. 4, we present our results and findings. Finally, in Sect. 5, we discuss design principles meant to provide guidance on designing XAI for trust calibration.

## Theoretical background and related work

The decision-making process in collaborative environments is based on conveying information between the collaborative members [61]. Decision-makers in such environments may need to process various information to make the final decision [5]. The modality in which the information is communicated between the members has a significant role in decision-making quality [30]. Also, the increase in the complexity of the decisions triggers an increase in the information needed to explain the underpinning logic and, hence, the effort needed for effective usage of the available information. According to [61], people at the time of making the decision are highly influenced by individual and affective factors of trust; some are related to the entity conveying the information, e.g. whether a human or a computer system.

In Human–Computer trust literature [42], two distinct trust dimensions are identified: cognition-based trust and affect-based trust. These dimensions distinguish between the cognitive components of trust from the emotional components. Cognition-based components include perceived understandability, perceived reliability and perceived technical competence. Cognition-based trust enables people to use their intellectual and reasoning skills. On the other hand, affect-based trust includes personal attachment and faith. These components of trust refer to the emotional bond, in our case, between the human and the AI, which does not result from reasoning and understanding but from feeling, sense and previous experience. Previous research showed that both affect-based trust and cognition-based trust have an impact on the decision outcome [97]. Cognition-based trust is crucial for establishing appropriate trust, whereas affect-based trust is developed as the relation continues [53]. Furthermore, previous research showed that in critical decision-making scenarios, it is highly likely that cognition-based trust components are more significant for trust calibration [41, 44].

Calibrated trust is the process of successful judgment of main components of trust: cognition-based trust and affect-based trust [40, 52]. People evolve their level of trust, in both dimensions of cognition and affect, considering the current state of the AI and their experience with it. As the underlying nature of AI applications is inherently uncertain and dynamic, decision-makers working with an AI face difficulty in calibrating their trust in an agent. The uncertainty and complexity may lead them to over-trust the system and follow an incorrect recommendation or under-trust in rejecting a correct recommendation. Previous research [40, 88] identified five primary contexts where trust calibration errors in automation occur, their reasons of occurrences and potential design solutions. These errors can happen when users do not understand the system functionality, do not know its capability, are overwhelmed with the system output, lack situation awareness or feel a loss of control over the system. Such faulty design has shown critical safety issues [68].

One goal of explainable artificial intelligence is to mitigate trust calibration errors [92]. The approach aims to help users of intelligent systems build an appropriate trust level by showing users the rationale and reasoning behind an agent recommendation. Although, many studies showed that explanation could indeed improve trust calibration [80]. However, such studies often assumed that users would engage cognitively with explanations and calibrate their trust. Recent studies showed that even though explanations were communicated to people, trust calibration is not improved [16, 96]. Such failure of XAI systems in enhancing trust calibration has been linked to factors such as humans’ cognitive biases, e.g., people are selective of what they read and rely on [57]. Also, others showed that XAI failed to improve calibrated trust because of undesired human behaviour with AI-based explanations, e.g., human laziness to engage in what they perceived as effortful behaviour [87]. Overall, users of XAI systems fail, on average, to calibrate their trust, i.e., human decision-maker working collaboratively with an AI can still be notably following incorrect recommendations or rejecting correct ones. This raises a question on how to design explanations to improve or operationalise trust calibration in XAI interfaces. This study aims to explore how XAI interface design can improve the role of explanations in calibrating users’ trust and enabling a successful judgment for trust components. Our deriving framework to discover protentional design solutions was digital nudging [20] and also the principles of de-biasing [75]. Testing whether our results would make that impact would require further testing, possibly within experimental settings. For example, we can hypothesise that a technique like nudging through shuffling the options can break through the status quo bias and make users more receptive to a different route of thinking, more reflective than automatic. This, however, will also depend on several variables and require extensive research to fine-tune. For example, personality traits like openness to a new experience [36] can play a role in people decision making and hence respond to nudging in the way mentioned earlier.

## Research method

Our study design and analysis of the data are situated within a two-dimensional space: *everyday Human-AI collaborative decision-making task where trust calibration errors are possible, and AI-based explanations to support trust calibration*. Through multi-stage qualitative research, we aim to answer the following questions:

- **RQ**: *How to design for explainability that enhances trust calibration? What design techniques could be implemented, and what are suitable principles to guide the design?*

To this end, the research method of this paper included two phases: Exploration and Co-design. The exploration phase aimed to explore how users of everyday Human-AI collaborative decision-making tasks interact with AI-based explanations and why explanations are not improving trust calibration. The co-design phase goal was to investigate how users of XAI systems would like to integrate AI-based explanations in their everyday decision-making task. Co-design phase helped us to understand how the solution would look from users’ perspective. The materials used in the two stages can be found in the published technical report [54]. The following sections describe the research method.

### Use case and underpinnings

**Figure 1:**
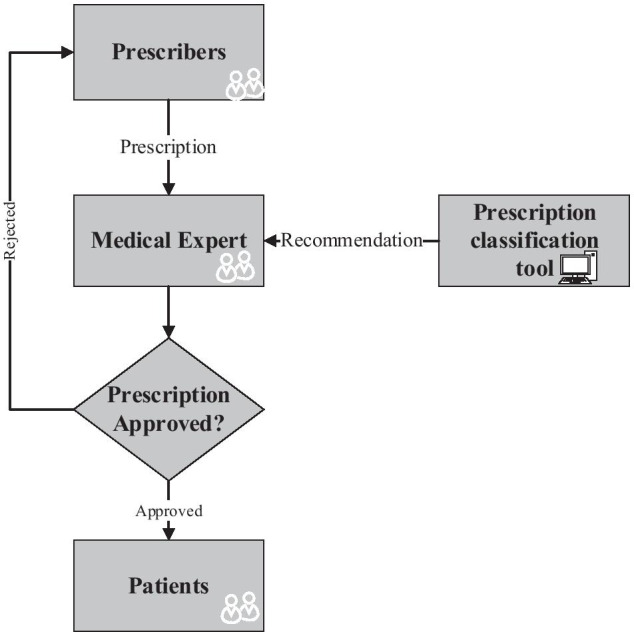
Screening prescription classification AI-based system classification

Screening prescription is a process that medical experts in a clinic follow to ensure that a prescription is prescribed for its clinical purpose and fit the patient profile and history. The main workflow of the prescribing system shown in Figure 1.

**Figure 2:**
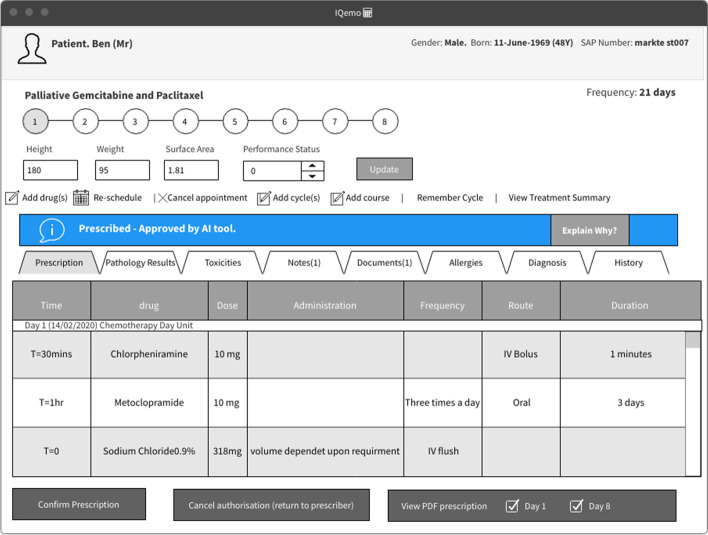
A sample of prescribing system interface supported with AI recommendations

To help our investigation, we designed an AI-based decision-making mock-up meant to help classify the prescriptions into confirmed or rejected. We chose this case study to reflect an everyday Human-AI collaborative decision-making task where trust calibration errors are indeed possible. We designed the mock-up based on templates and interfaces familiar to our participants in their everyday decision-making tasks (See Figure 2). Our mock-ups mimic a web-based tool and are meant to simulate the user experience when working on an existing system. As the medical expert clicks on a prescription, the tool shows the patient profile and the recommendation from the AI-supported decision-making tool (confirmed or rejected). The user has access to AI-based explanations to understand the AI rationale of why the prescription should be confirmed or rejected.

### Participants

We recruited twenty-four participants primarily through an email invitation. Sixteen participants were involved in the exploration phase and eight participants in the co-design phase. The email was sent to oncology departments in three organisations in the UK. We designed a pre-screening survey to get participants’ demographic information and their experience in screening prescription as this was the domain we choose for recommendations. Table 1 shows the demographic information of our participants. We anonymise the organisations with A, B and C for data confidentiality purpose. The inclusion criteria for both phases included medical experts who used clinical decision support systems before and have experience in the selected use case. We have chosen the scenarios and recommendations in a way they equally apply to all of our participants in terms of needed expertise and skills. However, the choice of a different role was motivated by the nature of the task, i.e., screening prescription Human-AI task can be followed by doctors and pharmacists in the clinic.

**Table 1:** Participants demographics for exploration and design phases

Phase	Participant	Gender	Age	Role	Year of experience	Organisation
Exploration	P1	Male	20–30	Medical Doctor	1–5	A
	P2	Male	20–30	Medical Doctor	1–5	A
	P3	Male	20–30	Medical Doctor	5–10	B
	P4	Female	20–30	Pharmacist	5–10	B
	P5	Female	20–30	Pharmacist	5–10	C
	P6	Male	30–40	Medical Doctor	5–10	A
	P7	Male	30–40	Medical Doctor	10–15	A
	P8	Male	30–40	Medical Doctor	5–10	B
	P9	Female	30–40	Pharmacist	10–15	B
	P10	Female	30–40	Pharmacist	15–20	C
	P11	Female	30–40	Pharmacist	10–15	C
	P12	Female	30–40	Pharmacist	10–15	C
	P13	Female	30–40	Pharmacist	10–15	A
	P14	Male	40–50	Medical Doctor	15–20	B
	P15	Male	40–50	Medical Doctor	15–20	B
	P16	Female	40–50	Pharmacist	15–20	C
Co-design	P17	Male	20–30	Medical Doctor	1–5	B
	P18	Male	20–30	Medical Doctor	1–5	B
	P19	Female	20–30	Medical Doctor	5–10	D
	P20	Female	20–30	Medical Doctor	5–10	B
	P21	Female	20–30	Medical Doctor	5–10	D
	P22	Male	30–40	Medical Doctor	5–10	B
	P23	Female	30–40	Medical Doctor	10–15	C
	P24	Male	40–50	Medical Doctor	15–20	B

### First phase: exploration

In this phase, we conducted a think-aloud protocol with sixteen participants. Think-aloud protocol helped to discover trust calibration errors in the context of explainability. Also, we aimed to explore why explainability is not improving trust calibration. We asked participants to use our AI-based decision-making mock-up tool and make decision collaboratively with the tool. We encouraged participants to think aloud while viewing and reading the explanations and making decisions. Each think-aloud session included ten Human-AI collaborative decision-making tasks. The Human-AI task included a patient scenario, an AI-based recommendation, an explanation, and the participants’ role was to accept or reject the recommendation. We developed the tasks to be non-trivial and included correct and incorrect AI recommendations based on external medical expert judgment. This ultimately helped put our participants, who were medical experts, in a realistic setting: exposing them to an imperfect AI-based recommendation and its explanations where trust calibration is needed and where errors in that process are possible. We tested the material and tasks with two participants and refined them to optimise their fulfilment of these criteria. Finally, the ten Human-AI tasks performed by participants varied in their explanation types. We used five different explanation types revealed from our literature review. The material used in this stage can be found in [54]. Below, we provide a brief description for each explanation type:

1. Local explanation: This type provides explanations at the AI-based recommendation level; this could be either by quantifying data features contribution values or generating rules and decision tree for a particular recommendation [67].
2. Global explanation: explanations of this type provide the general logic and rules of AI reasoning [90]. This includes presenting weights of different data features as decision trees, set of rules or ranking style.
3. Counterfactual explanation: explanations of this type quantify a situation where the AI could change its recommendation [86], e.g., the patient would not develop cancer if his years of smoking < 5.
4. Example-based explanation: explanations of this type present examples of similar cases in the dataset [17], e.g., patient A would develop cancer because he has a similar profile to patient B.
5. Confidence: explanations of this type shows an algorithmic certainty of the recommendation which reflect a probabilistic chance of a correct recommendation [96].

**Figure 3:**
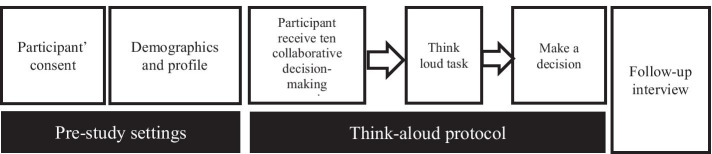
Exploration phase workflow

Using five explanation types in our study was meant to eliminate potential bias in our results being relevant to only certain type of explanation. This also helped us to trigger different responses from the participants. For the data analysis, we considered a trust calibration error when it happened across all explanation types. Future studies such as controlled experiment will be needed to map between trust calibration errors and explanation types. At the end of this stage, follow-up interviews were used to clarify the collected data and gather insights from the participants about their lived experience with AI explanations. Each think-aloud study lasted for around 50–60 min. Figure 3 summarises the Exploration phase workflow.

### Second phase: co-design

We conducted two co-design sessions with eight participants, i.e., four participants in each session. The main aim of this stage was to explore how the design can play an effective role in enhancing users’ trust calibration during a Human-AI collaborative decision-making task. We used the same inclusion criteria employed in the exploration stage, i.e., expert users in the Human-AI task. We chose to recruit different participants to avoid the learning effect [39] and increase the credibility of our findings as existing users already learned the objective of the study and were part of the underpinnings for this next study. Co-design method enables users who might be potential users in future AI-supported decision-making tools to reflect their experience in the design process, and this is supposed to increase the acceptance of the proposed solutions [66]. Co-design can lead to a better understanding of the end-user needs, which enhances the possibility of the designs’ acceptance [76]. In this phase, we discussed and negotiated how to embed AI explanations to serve users’ needs, task workflow and trust calibration. Together with the participants, we conceptualised and sketched design features to support users in utilising AI explanations and reduce trust calibration errors revealed from the exploration phase. This was achieved by allowing the participants to use and amend our AI-based tool or mock-ups. This method is useful to help them visualise the idea and then provoke brainstorming related to the research problem [22]. All these dynamics were hard to capture during the exploration phase. Therefore, co-design method helped us to develop innovative designs of how the solution should look from a user perspective.

Participants were divided into two design sessions based on their availability. Due to the COVID-19 situation, we chose to conduct the study online using FreeHand tool from Invision.^1^ Also, it has been shown that online tools for co-design can make the process easier, cheaper and flexible for participants [58]. To mitigate any potential issues that could arise from using online platforms, e.g., readability of the instructions and the tool usability issues, we conducted a pilot study with two post-graduate researchers and one academic in an interdisciplinary research group residing in the departments of Computing and Psychology in Bournemouth University. This also helped us in the preparation of the training and induction stage for the participants in the real study. Then, participants were invited to training session to familiarise themselves with the tools’ functionalities and how they can communicate online. The training session lasted for 15–20 min. Participants were also invited to try the tool till they felt all capable of using it. They had the ability to ask questions and one of the authors answered them.

**Figure 4:**
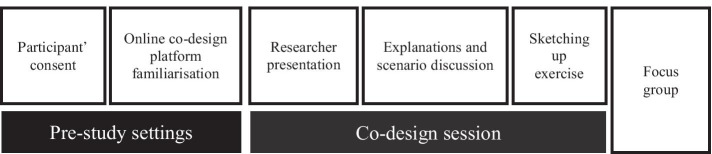
Co-design session workflow

We adopted four techniques during the co-design sessions in order to reach the goal of our study (See Figure 4); researcher presentation, participants discussion, sketching-up exercise and focus groups. This also helped to enhance the credibility of the study and to ensure that data bias was eliminated. Each of the sessions lasted for around 2 h. Both sessions, including the four main steps, were audio-recorded and transcribed. Audio recording for the design session helped the authors analysing main design needs and issues revealed from participants discussions. The following sections describe each technique that we used in our design sessions.

1. Researcher presentation (10 min). The researcher gave a 10-min presentation on AI-based decision-making tools and an overview regarding the first phase findings, particularly those about different types of errors that emerged during the exploration study. This helped to immerse the participants in the research problem, and it involved a warming-up activity in getting the participants involved in the design sessions.
2. Explanation and scenario discussion (25 min): In this stage, participants started by introducing themselves. We then asked each participant to talk about how AI-based tools could help their everyday decision-making process. Then, we provided a definition for explainability methods introduced in previous interpretable machine learning surveys [2]. We also provided different e-cards describing different explanation types in simplified examples. This was meant to illustrate explainability definition and potential uses of these explanations. To answer our research question, the participants needed first to immerse in a fictional problem as recommended in [15]. In our study, the fictional problem was collaborative decision-making between the medical expert and the AI. Specifically, a screening prescription using an AI-based tool. We asked our participants to use screening prescription tool and examine how they would like to receive its explanations. This stage was meant to scope the discussion and facilitate focused conversations. This was also meant to immerse the participants with the research problem and facilitate their understanding of the researcher presentation. Our participants discussed a wide range of trust calibration scenarios using the provided material in this stage. This stage provided a sense of realism to the problem and encouraged careful consideration of solutions to cater to different contexts and usage styles.
3. Sketching-up exercise (40 min): Participants were then encouraged to start sketching up their designs using FreeHand tool from InVision. We gave each participant a blank e-page to sketch up designs. The online platform provided several creation tools (e.g., coloured pens, shapes and sticky notes). The participants were also asked not to limit themselves to the given explanation and consider any extra features they would like to see in XAI interfaces to help them utilise the explanation during a collaborative decision-making task. We deliberately asked our participants to work individually, think outside of the box, and consider different kinds of potential solutions. In this stage, our participants designed their explanations and provided multiple usage scenarios for them. They created a wide variety of usage scenarios covering different purposes and task requirements, e.g., grouping data features in Local explanations to reduce the explanation complexity.
4. Focus group (45 min). After each participant completed the sketching activity, each participant presented their ideas to the group. This was meant to critically analyse and evaluate the ideas by the participants to formulate robust solutions. In addition, this activity allowed our participants to explore and discuss various ways of using AI explanations in their work environment, considering trust calibration as the primary goal.

### Data analysis

Our data were collected across two stages: Exploration and Co-design. First, we analysed the collected data from the exploration stage, including participants’ audio-recording transcriptions and researchers’ observations and notes. The analysed data included themes for trust calibration errors that need to be addressed in the XAI interface. Exploration analysed data used as an input for the co-design phase. Second, we analysed the data collected during the co-design phase, including audio-recording transcriptions, sketching data and researcher notes. Both stages were analysed using content analysis. Content analysis is used to search for themes and concepts that emerge concerning the study problem. We first familiarise our self with the data. Then the first author coded the data. The other authors mentored the process and verified both the coding and the conclusions made. We followed an iterative process across several research meetings to formulate, combine, and conceptualise the emerged concepts. This iterative process was meant to examine and ensure the codes were interpreted and assigned to the correct themes.

## Results

While a considerable effort has already been made to improve the accuracy of the explainability models and their fidelity with the underlying algorithms [2], our research contributes with a new understanding on how to design for explanations that support calibrating trust. We investigate the critical role of integrated, coherent and relevant explanation design for calibrating trust. We analysed our participants’ designs and their transcriptions and focused on major trust calibration factors that emerged in the study phases. In the following sections, we describe the emerged themes revealed from each of the study phases.

### Exploration phase

While participants used our AI-based tool to perform a Human-AI collaborative decision-making task, many participants made systematic errors during their interaction with the explanations. They either skipped the explanations or misapplied them in their decision-making task. In this section, we describe how participants interacted with AI explanations during their collaborative decision-making task. We focused our analysis on situations when participants failed to utilise the explanation, and that led to trust calibration errors. We provide a summary of the analysis in the following paragraphs and Table 2. The full analysis is published in [54]. This summary will be useful to understand some of the trust calibration issues as they can relate to the interaction with the explanation and attitude towards it in principle, besides its informational content and delivery method.

**Table 2:** The main types of errors made by participants when interacting with explanation interfaces

Error type	Sub-category	Description
Skipping	Lack of curiosity	Participants had a lack of desire to know, learn or experience an explanation. They did not feel that the explanation motivated them to learn new ideas, resolve knowledge and assist them in the task
	Perceived Goal impediment	Participants perceive the explanation as an interruption to their decision-making task and delayed their task goal
	Redundant information	Participants skipped the explanation because they felt it was repeating facts, and it did not add substantiations
	Perceived complexity	Participants skipped long explanations, as they did not want to engage in what they perceived to be an effortful experience
	Lack of domain context	Participants skipped explanations that were not reflective and contextualised to their domain knowledge context
Misapplying	Misinterpretations	Participants misunderstood the explanations which lead to incorrect conclusions
	Confirmatory search	Several participants searched for information that confirms their initial hypothesis, i.e., they were selective in what to hear and rely on
	Rush understanding	Participants incorrectly held a belief that they understand the AI deeper than they actually did, i.e., they failed to recognise the limits of their own understanding
	Habits formation	Participants became gradually less interested in the details of explanation and overlooked and perceived it to be familiar to them
	Mistrust	Participants felt that explanations were not reflecting knowledge, or they were suspicious, i.e., they voiced scepticism about the correctness and validity explanations

#### Skipping the explanations

We observed that participants made decisions collaboratively with an AI-based decision-support tool without thoroughly reading the explanations. As a general theme, when we debriefed participants in a follow-up interview, we found that participants did not always remember the explanations that they had skipped. For example, P3 later could not remember the Global explanation, which she skipped while making a decision. In contrast, P14 skipped the Local and Counterfactual explanations but still remembered their general insights. Another example of skipping the explanation was when P5 felt the explanation will not add value to her current knowledge. P5 mentioned, *“… to be honest with you, I was not really interested in reading the explanation … I mean I did not feel that could add something new to me*”. We categorise participants skipping behaviour into five categories: Lack of curiosity, Perceived goal impediment, Redundant information, Perceived complexity and Lack of context (See Table 2). As we elaborate further in the Discussion section, this finding raised the question about the extension to which people engage with the explanation and how to combat their avoidance if needed.

#### Misapplying the explanations

Even though participants interacted with the explanations and made their decisions based on these explanations, we observed several participants misapplied the explanations in their decision-making task. Participants had a good mental model about AI reasoning and its explanations, but it was not complete which led to misapplied behaviour. As a general theme, we observed that participants needed adequate information and aid in the interface design to support them in optimising their explanation usage in their task. For example, participants were sceptical in adopting the explanation in their decision-making task due to concerns regarding the data source and the validity of the explanation. This lack of transparency led to participants not trusting the provided explanation, and therefore, not achieving the goal of explainability to facilitate trust calibration. P8 noted, “*I am wondering if an experienced pharmacist has looked at this before*”. Another example of misapplying errors when several participants became gradually less interested in the details of an explanation and overlooked it altogether. P4, who showed similar behaviour, mentioned, “*I think this is similar to the previous explanation*”. We propose later in the Discussion section a set of design principles, such as training and design for friction, to support help users’ trust calibration in such cases. Table 2 summarises situations in which participants misapplied the explanation, leading to trust calibration errors while performing a decision-making task.

### Design phase

Our results showed that users view explanation interfaces as a new interactive system that needs to be customisable to their needs and task workflow. Across the design phase stages, we identified four main themes characterising Human-Explanation interaction: *abstraction*, *cues*, *control*, and *adaptation*. These categories help to illustrate different explanation interaction and presentation techniques that need to be considered during the development phase of explainable interfaces. They also can be considered as high-level design requirements to support users in utilising the explanations during Human-AI task. Our proposed design techniques are not mutually exclusive as the explainable interface design could contain one or all of them based on the nature of the task and the XAI model. In the following sections, we present four categories of such design techniques that resulted from our analysis. Table 3 provides a summary of the suggested designs during the co-design sessions and their definitions.

**Table 3:** The four main themes that emerged from the co-design phase

Design technique	Definition
Abstraction	It refers to extracting and generating main features from the explanation and make it possible to present them at multiple abstractions and granularity levels
Control	It refers to providing customisation functionality to control the information presented in the explanation (e.g., grouping, ordering)
Cues	It refers to additional elements that can draw users’ attention and help guiding them in the process utilising the explanations
Adaptation	It refers to varying the explanations characteristics, e.g., information, abstraction level, cues, order and modalities, in response to an interaction context, i.e., the ability to communicate explanations differently in different settings

#### Abstraction

**Figure 5:**
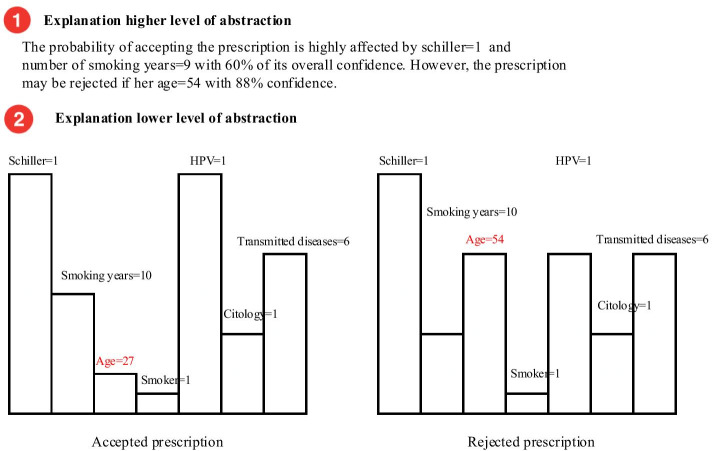
An example of two levels of abstraction design presented in our co-design study

Abstraction design technique refers to extracting and generating main features from the explanation to be presented at multiple abstractions and granularity levels in the XAI interface. Abstraction is intended to make it easier to read an explanation and recall the meaning at an appropriate level of details for the user profile and expertise and interest. Numerous studies have shown that the amount of presented information affects users’ decisions quality, cognition and trust [65, 69, 71, 79]. Several participants sketched up explanations that had an abstraction feature according to their ability to understand the explanation and their interest in the details. P19 described his design as “*in this way, I can easily know and remember what is happening*”. One of the produced designs that implemented an abstraction technique can be seen in Figure 5. In this design, P22 combined Local and Counterfactual explanations into one interface and reduced them into two levels of abstraction. The higher level of abstraction contained a narrative summary for Local and Counterfactual main characteristics. The next level of abstraction was to observe in detail the two explanations and compare them together. Participants seemed to be interested in multiple levels of details to minimise the possibility of becoming overwhelmed during their decision-making task. P23 commented on the abstraction design feature: “*This is quite useful … it is good to have different levels of details … sometimes I do not need to look at all patient information*”. Other participants also agreed on this sentiment: “*it is the quickest and easiest to see at a glance the information you want*” and “*It is informative but also easy*”.

In general, when an AI explanation was presented to our participants, they sought to simplify the explanation into multiple levels of abstraction. In psychology, humans process information at a different level of abstraction to cope with complexity and optimise cognitive effort [24]. For instance, people usually start reading the main title of the article and then the headlines and then, potentially, the article text. Our participants were more inclined to spend more time on a specific abstraction level more than reading the explanation as one chunk. Abstraction design has been widely accepted and implemented in human–computer interaction [73]. Te’eni et al. (2005) showed that people usually focus on a particular level of information at a given stage of the decision-making process.

In summary, the abstraction design technique can be utilised to facilitate users’ concentration on a particular level of explanation abstraction and then shift to another level. It also could increase users’ engagement with a usable and user-friendly explanation. For instance, decision-makers may not need to go through and process all the abstraction levels to understand the current recommendation and the explanation in cases familiar. This technique could be implemented at the design level using colours, fonts, folding-unfolding technique and multiple views. Designers of explainable interfaces may need to consider breaking the explanation into various levels to help users better understand, even for complex decisions and explanations. Also, understanding when and under which conditions the users shift from one level of abstraction to another helps produce more effective trust calibration explanation interfaces. We need further research to design the abstractions level and the navigation between them on the one hand, and the trust calibration process on the other. For example, a question to ask is whether viewing an abstraction at only a higher level of abstraction means a flawed trust calibration process and whether familiarity with the case can always be seen as a moderating factor.

#### Control

**Fig. 6:**
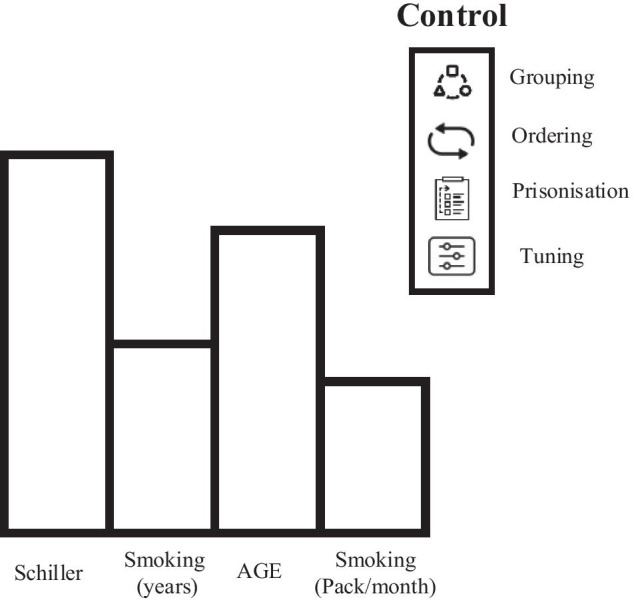
An example of suggested control techniques designs for Local explanations in the co-design sessions

Participants discussed the need for control functionality that allows them to contextualise and personalise the explanation content. These observations are consistent with trust research that showed trust could be developed inappropriately when the explanation does not match the user experience and expectations [40]. Also, recent research showed that static explanations often fail to provide a satisfying explanation among all users [74]. In the following sections, we present a group of control techniques proposed during the design sessions and meant to enable the generation and delivery of a contextualised and personalised explanation and, ultimately, help a better trust calibration process (Figure 6).

##### Ordering

It refers to enable controlling the order of the explanation content. Participants discussed that such function would meet their user experience and reasoning process, e.g., P17 stated: “*Well, usually I would look for patient age, smoking habits and history … It would be more beneficial to see this information first*”. Several participants sketched different orderings of explanation types and explanation content in their designs in which the order met their decision-making strategy. This pattern also emerged in the focus group stage when participants discussed the role of controlling the order in their decision-making strategy, e.g., P20 mentioned, “*I think this is a subjective thing … some practitioners may need to look first for transmitted diseases*”. Order effect is a well-studied phenomenon in the literature of behavioural decision-making. It studies the influence of the information order on decision-making outcome in a more or less systematic fashion [77]. The information offered at the beginning of a sequence might be more influential when people make decisions, an effect known as primacy [83]. Also, recency refers to the significant influence the information presented at the end of a sequence has [9]. Whether primacy and recency, this effect of information ordering have been experimentally validated in decision-making environments [25]. In human–computer interaction literature, controlling the order is considered an effective technique to meet the evolving user experience [31]. Similarly, in collaborative AI-based decision-making tools, decision-makers trust judgments are based on several explanation types and information presented in the explanation. The order of an explanation in the XAI interface or a piece of information in the explanation could affect users’ decisions, e.g., an explanation can be designed to tell the influence of patients’ age before patients’ smoking behaviour on AI cancer prediction or put them together in the same order.

##### Grouping

With the large numbers of data features that could be presented in explanations such as Local and Global explanations, our study showed a trend where six participants grouped a set of relevant features to calculate group-feature importance. P19 mentioned: “*It makes more sense to have a value represents the importance of the transmitted diseases*”. They argued that grouping similar data features in the explanation could reduce its perceived complexity and enable more contextualised and informative explanations. P21 stated: “*… doctors sitting in the clinic will see that complex. Gathering all patient smoking information in one value is more informative*”. Our participants suggested two ways of grouping the features: *default groups*, e.g., patient history, laboratory tests, and *user-created* grouping, i.e., ability to create a group, e.g., features that the user thinks are relevant to each other. Our results are consistent with the previous survey by [11] who found that enabling the data feature grouping is vital for non-AI experts’ understandability. Such a technique was essential to our participants to contextualise the explanation in their environment and assess its correctness. P22 commented: “*… combining all the patient history values together makes more sense and helps to draw proper conclusions*”. Given this feedback from our participants, it would be essential to allow participants to amend the way that they would like to present the AI explanations, mainly in grouping some data features to convey meaningful interpretation from their perspective.

##### Prioritisation

It refers to enabling users to prioritise the explanation interface components based on the users’ perspective; this includes choosing their preferred *explanation type* and *explanation content*. Participants argued that this feature would help them in reducing the complexity of the explanation interface and tailoring it to fit their reasoning process. For *explanation type prioritisation*, we observed that several participants used different explanation types in their designs and ignored the other explanation types. For instance, P19 centralised her design on Local and Example-based explanation. P19 stated, “*In my opinion other explanations are complementary information*”. P17, on the other hand, wanted the explanation interface to include Local and Counterfactual explanation. The discussion about the expressive power of explanation types and disagreement about it, made our participants suggest including an explanation type prioritisation feature where users can choose their preferred type. On the other hand, participants discussed two scenarios to prioritise the *explanation content*: *manual* and *automatic* prioritisation. Manual prioritisation can be seen in P22 Local explanation design when he mentioned: “*I do not need to look at all these patient data effects on AI decision, five or six important ones should be enough … it is good to have a feature to choose and prioritise amongst them*”. Furthermore, automatic prioritisation was discussed when participants were overwhelmed with the number and explanation size. Automatic prioritisation was mainly about setting thresholds for the importance of the explanation content to be presented in the interface with respect to the AI recommendation. For instance, two participants in Global explanation wanted to show the features which had at least a 15% impact on the AI reasoning. P24 stated, “*I only need to see reasons that have a 15% or more influence*”. Previous research explained this result when they showed that the explanation interface's complexity varies between humans and affects willingness to interact with the explanation [12, 50, 59]. Some users prefer a detailed explanation, while others need a brief explanation based on their curiosity level [47]. Overall, allowing users to guide and customise the explanation interface might benefit trust calibration goal by making the interface output more suitable to the users’ experience and the reasoning process. However, there is also a risk that this freedom of choice leads to a biased trust calibration process. Balancing between the user experience and the goal of calibrating trust objectively is a question to address in future research.

##### Explanation tuning

It is a control feature in which users can tune or change specific explanation engine parameters or configurators to generate different explanation instances. We observed that lack of verification techniques had motivated our participants to propose serval designs with a tuning functionality. P18 stated: “*Well … it is not fair for an AI to explain in this way … it would be useful to try different scenarios from explanations*”. As a general theme, the explanation output was not satisfying to our participants. They required an interactive and verification technique to motivate them in using the XAI interface. Our study identified three categories of explanation tuning:

1. *Degree of similarity.* This category was witnessed in Example-based explanation. It refers to tune the example similarity to a preferred threshold. For instance, P17 commented on his design: “*In medicine, it is hard to define similarity. I would like to define it and amend it so I can judge the explanation in a better way*”.
2. *What-if analysis*. This category was witnessed in all explanation types. It refers to allowing users to ask what-if questions and directing them to verify the recommendation. In one scenario, when counterfactual explanation explained a recommendation of a patient to reject the prescription: “*The prescription would have accepted with 67% confidence if the smoking years* = *15 and Hormonal Contraceptives (years)* = *13*”. P21 asked, “*Could the AI ignore Hormonal Contraceptives and re-explain how the prescription could be accepted*”.
3. *Time frame.* This category appeared in all explanation types. It refers to a technique to regenerate the explanation output based on data gathered in a specific time frame. Several participants discussed their concerns regarding the effect of outdated data on the explanation. For instance, in Example-based explanation, P20 asked to regenerate examples excluding data before 2010 as old examples might be misleading, e.g., healthcare and lifestyle change rapidly, and timely data about this is essential to judge the explanation and recommendation it explains.

Participants requests served a need for an interactive technique to help them in guiding the explainability process and meet different thresholds of their recommendation judgement. Our results are consistent with human-automation interaction literature which identified users’ input into the intelligent system as an essential requirement for achieving trust calibration [40, 74]. They argued that users’ involvement could be a crucial in the trust calibration process, especially when the underlying reasoning is dynamic and changing over time.

#### Cues

Cues refer to additional elements in the interface that can draw users’ attention and guide them in understanding and reading the explanation [3]. Users interact with a large amount of information every day and may occasionally fail to recognise and detect important details in the explanation. Also, in the long-term, explanations might become peripheral or checking them becomes habitual but not necessarily conscious and effective in drawing the users’ attention to important details and nuances [85]. Our participants sketched interfaces with two cues categories (visual and information) to guide them in the process of reading and quickly judging the explanation. In the following sections, we present two categories of visual cues that resulted from our study.

##### Visual cues

Colour coding, font coding, shape coding, and directional cues were among the visual techniques used to help quickly read relevant information. Participants used visual cues to focus their attention on relevant information in the explanation interface and guide them in utilising it. For example, our participants used green/yellow/red colour schemes for faster perceptual judgment of the Confidence explanation value (“high, “moderate” and “low”). Another example when participants’ sketches for Local and Global explanations revealed visual cues aligned with data features to provide more contextualisation and understandability. Three participants used a red/green colour scheme with a local explanation to distinguish between data features that positively influenced the recommendation (green) and those that negatively influenced it (red). Participants in Counterfactual explanation designs also used directional cues such as arrows and pointers to point out the direction of the changed data feature, i.e., in either increasing or decreasing the changed data feature value. In HCI literature, visual cues have been used to reduce overlooking specific part of the interface for high critical tasks [21, 62]. Overall, visual cues could be an assistive design technique for explanation utilisation. It would help quickly understand the explanation and direct the user to different sections in the explanation interface. It aids their trust calibration process by finding the relevant information and reducing fatigue and time needed. Visual cues should be defined in the design process with collaboration with end-users and HCI expert to identify key visual cues to be added to parts of the explainable interface.

##### Informational cues

It is an indicator of the explanation quality. Decision-makers in uncertain and dynamic environments might lack the ability to process all the available information, e.g., explanations, so they use informational cues to assess its quality before reading it. Informational cues have used in the literature as a signal of information quality [32] and increase users’ ability to detect errors [8, 88]. In the context of our study, participants used multiple information cues in their explanation designs to help them in judging the explanation quality and identify the extent to which they can trust it. P21 discussed using a timestamp informational cue with Example-based explanation and stated, “*doctors cannot use these examples in this way … they would need to see when each example case has been prescribed”.* Participants discussed that they would be more willing to read the explanation when it can show its own quality. For instance, P24 used an expert validation informational cue with Local explanation to indicate that the explanation has been designed with medical expert validation. P24 mentioned, “*I thought this [informational cue] would be handy to make people trust the explanation*”. Likewise, P17 integrated an informational cue to the interface to inform about potential missing information in the patient profile, which might lead to an incomplete explanation. P17 argued: *“… in this way I would know that the explanation is incomplete*”. This is a common approach used in high-stakes decision support systems to increase the safety, speed and accuracy of the decision-making [21]. Our findings are consistent with previous research in information processing to build trust [95] showing that people subconsciously look for informational cues when deciding whether they can accept the presented information or reject it. Consequently, exploring additional informational cues for each explanation could enable better users’ assessment explanation quality and facilitate effective explanation utilisation.

#### Adaptation

Adaptation refers to varying the explanation characteristics based on the interaction context. Adaptation is itself an intelligent decision and requires the development of the variability space of explainability options that are paired, either directly or through following some inference rules, to the task and personal states. As a general theme, participants felt that a well-designed adaptation technique could increase their perception of the explanation competence and encourage them to utilise the explanation. P11 stated: “*AI should be smart enough to know when to explain and how to explain*”. We noticed two categories of explanation adaptation characteristics among participants' adaptation designs: interface complexity and interface content order.

- *Interface complexity.* It refers to varying the complexity of the explanation interface to meet the collaborative decision complexity. P19 and P20 discussed situations where increasing the decision complexity should trigger the need for a higher level of details in the explanation interface. P19 mentioned: “*I think it is unfair to explain this way, AI should consider presenting sophisticated examples and correlations in such complex case*”. Then P20 added: “*indeed … also with some patients, I don’t need that much information to determine if the AI is right or wrong*”. Moreover, participants discussed varying the level of explanation interface complexity based on the AI confidence, interestingly, they expressed a low level of trust in this case, P5 described: “*I would trust the AI more when it is not certain and provide a short explanation or just say I cannot explain … not enough evidence*”. Overall, our participants expected the AI-based tool to understand the decision complexity and generate an appropriate explanation.
- *Interface content order.* It refers to an intelligent decision to change the order of the explanation type or explanation content based on the decision context. Several participants discussed situations that required a variation in the *explanation type* order in the explanation interface in different contexts. For instance, P24 stated that when the patient has Human papillomavirus viruses (HPV), Example-based explanation has a higher impact on the decision. P24 stated: “*HVP is the most important risk factor for this type of cancer some of them can cause a type of growth called papilloma … in such cases, I usually look for the similar patients first”.* Our analysis also showed a pattern where three participants discussed scenarios for *explanation content order* adaptation. P20 discussed presenting the smoking effect in Local explanation first when the patient has a smoking habit. P20 mentioned: “smoking *is a high cause of accepting or rejecting such treatment when a patient does have that … it should be at the top of the graph*”.

Our results are consistent with previous research [45, 94] which showed that transparency that is in a conflict with the user experience and requirements of the task at hand might trigger cognitive confusion, mental overload and trust calibration errors. This means, explanation alone is not enough but rather its delivery and presentation should adapt to the user and task context to avoid that conflict. Also, our previous literature review revealed that explanation shall be adapted to users’ level of knowledge and expertise in a given Human-AI collaborative decision-making task [56]. Similarly, previous studies showed that adaptive information increases collaborative Human–Robot performance [82]. Overall, the developers of explanation interfaces might require applying usability techniques like task analysis [51] to determine different contexts and scenarios where adapting explanation is required. Incorporating task characteristics and contexts into the explanation delivery methods would likely increase the efficiency of explanation utilisation, and thus, improve trust calibration. Adaptation with XAI interfaces can also be used to meet the continuous development of the users’ experience and meet their learning curve about the AI.

## Discussion

Human-AI collaborative decision-making tools are usually built to support trust calibration through the recommendations interface design [10, 35, 70]. For example, users are typically enabled to control the level of assistance they want to get from an intelligent agent, so it provides important benefits to trust calibration, including improved situation awareness and more accurate Human-AI performance [48]. This section expands the previous research by discussing five design principles that consider the interaction between the user and the XAI interface for improved trust calibration. We derived these principles as a reflection following a series of qualitative studies. This included the previously described studies of think-aloud and co-design sessions. The active involvement of XAI interface users and their lived experience facilitated identifying novel, interactive design concepts that would develop XAI interfaces that help trust calibration. As a conclusion of our qualitative studies, we provide more ecologically valid support for previous work on Human-AI collaborative decision-making, specifically when explanations are introduced. We tie our findings into a border discussion and principles on designing XAI interfaces to help trust calibration in the following sections. Although our principles provide guidelines for designing XAI interfaces to help trust calibration, calibrated trust XAI interface design may require a continuous improvement and monitoring in which the design is fine-tuned according to the feedback from the user, whether its explicit from the users or implicitly through behaviour indicators, such as a user is skipping explanations all the time. Hence, the XAI system will be able to infer the correlation between the required techniques, the explanation and the recommendation, which by time can become principles and lessons learned.

### Design for engagement

During our Exploration phase, several participants skipped the explanation presented to them due to factors such as lack of curiosity and motivation. Participants did not feel that the explanation motivated them to learn new ideas, resolve knowledge and solve problems. Also, for participants who were interested in reading the explanation, they applied heuristics to interpret the explanation in their task. These findings provide a possible interpretation for why exposure to explanations did not improve trust calibration [16, 96]. Furthermore, during the design phase, participants sketched up explanation structures that motivated them to read the explanation, e.g., abstraction and grouping. They were more motivated to engage with an explanation structure representing a reduction principle of the Persuasive Systems Design model [81], i.e. structures that present information in pieces and stages.

These results can be interpreted according to the Elaboration Likelihood Model of persuasion [63]. People follow two cognitive processing pathways: the peripheral route, which is fast and automatic and the central route, where people follow a slow and deliberative approach. People in everyday decisions tend to follow a peripheral route that employs heuristics and shortcuts to make decisions [37]. This can limit the role of XAI to help trust calibration as engaging cognitively with AI-based explanation is triggered rarely. Such behaviour might be the main reasons for skipping or misapplying the explanation. Indeed, successful trust calibration would require users to think and cognitively engage with the explanation. Therefore, an effective calibrated trust design may require XAI interface designers to increase users’ tendency to engage with the explanation and trigger the central route cognitively. Such engagement is determined by individual factors such as the need for cognition or peoples’ interest in the information [64].

A number of approaches can be followed from other domains to engage people with the provided explanations analytically. One of the promising ways appears in applying the principles of herd theory [6]. Herd cues can support users’ engagement with the explanation by providing information about other users’ interactivity actions. For instance, a herd cue message can be represented as the following “*several experts have used the explanation to judge the AI recommendation*”. Herd theory suggests that individual engagement will be influenced based on other actions in imitation-based behaviour [78]. Understanding users’ herd behaviour plays a critical role in influencing users’ engagement in information systems and can be effective in promoting a desired user behaviour and interaction style [84]. For instance, Barlow et al. (2018) showed that a herd cue message about the compliance of other users in changing their passwords increased the likelihood that users would comply with the same behaviour. The messages are to be framed carefully to avoid a situation where users become more inclined to follow the recommendations instead of following the behaviour of others of utilising the explanation to calibrate trust.

### Design for challenging habitual actions

A series of similar former responses might form habitual actions [60]. Habits can be triggered by environmental cues, such as time of the day; by internal states, such as individual mood; and by series of interaction with the same partner. Habits reduce sensitivity to minor changes in the explanation interface, curtail explanation utilisation, and reduce assessment and reflection about the decision [23]. For example, pharmacists who are in the habit of making decisions using AI-based screening prescription tool may fail to recognise incomplete explanation [72]. Previous experiment [29] exposed participants repeatedly to pictures of people to form well-practised reactions toward them. Participants were subsequently given the same pictures again but in a slightly amended version. Participants who had seen the faces repeatedly in the first part of the study had higher difficulty identifying the amendments and relied on their expectations formed during the prior exposures. These findings suggest that people with strong habits hold trust and expectations about the environment, which reduces their capacity to utilise the explanation and calibrate their trust.

During our co-design phase, participants frequently discussed taking an active role in controlling the explanation output to meet their reasoning process of everyday decisions – including the presentation and phasing, e.g., P12 mentioned: *“… I would like to exclude patient age from future explanations*”. Although such techniques could help users in their trust calibration and meet their user experience, however, they might be prone to develop habitual actions, which results in a failure in utilising explainability for trust calibration goal. Furthermore, in the exploration phase, several participants were gradually less interested in the explanation details and started to overlook the explanation. Hence, an effective design for a calibrated trust may need to consider challenging users from developing habits with the explanation interface. Research in psychology suggested two different habits challenging approaches which are *downstream* and *upstream* approaches [85]. The downstream approach focuses on the individual level of intervention and adopts strategies such as education, stimulus control and other behavioural modification strategies. One example of applying a downstream approach in XAI interface could be through developing educational material to show the benefits of explainability in calibrated trust. Educational material can also be used to inform users about the costs of undesired behaviour with the explanation and increase self-efficacy to perform the desired behaviour. In contrast, the upstream intervention approach targets more extensive structural conditions where peoples’ behaviours are embedded. For instance, upstream approach might change the structure of the XAI interface where the explanation is presented before the recommendation. This approach aims to provide a structure that promotes desired behaviour, i.e., presenting the explanation before the recommendation would increase the likelihood of reading it. These interventions in psychology literature gained their effectiveness because it renders people with strong habits open to new information [89]. We recommend future work on designing XAI for long-term explainability to focus on user experience for enabling usable explanation utilisation but also combat developing habits using approaches like the downstream and upstream intervention methods.

### Design for attention guidance

In both phases, we observed that participants needed the XAI interface to support them in reading the explanations. During the exploration phase, participants felt that the explanation was complex, and they were selective in what to read and rely on. This finding can be interpreted by the fact that human visual perception is selective [26]. Participants focused their attention only on a little number of elements in the interface and those only small portions of explanation content were processed. Such observations motivated our participants, in the design phase, to suggest design techniques and requirements to support them in reading the full explanation in a usable and user-friendly way, such as abstraction and visual cues. Our results from both phases suggest that helping trust calibration in the XAI interface design could be further enhanced by applying the principles of attention guidance [4]. The role of the attention guidance principle could be critical in the trust calibration process when users’ desired behaviour is to look for relevant content in the explanation and combat overlooking it. Utilising the explanation for the trust calibration goal would also expect to guide the user attention from one element to another and supporting them to determine the next element in the XAI interface. The main goal of such principles is to increase the amount of the processed explanation content by the users and thus improve their understanding of AI reasoning. Such a principle has been widely used in learning environments to help learners develop an improved understanding of the presented information [13, 26]. When designing attention guidance, a careful consideration shall be paid to whether the guidance itself lead to biased processing and becomes persuasive and lead users to neglect or follow the recommendation.

### Design for friction

Across the Exploration phase, several participants who worked collaboratively with an AI were more interested in the decision-making task itself rather the reading and understanding the explanation. This can be seen in categories such as perceived goal embedment and rush understanding. Participants developed a negative attitude towards explanations perceived to distract them from their main task. Moreover, participants frequently discussed or designed interfaces to accelerate their task completion time, e.g., abstraction designs. These results could be interpreted as avoidance or refusal behaviour. Our observations support previous work which found that people working collaboratively with an AI agent are not willing to engage in what they perceived effortful behaviour with the AI explanations [27, 28]. Overall, during both qualitative phases, the degree of willingness to read the full explanation was low, specifically when participants discussed using the explanation for their everyday decision-making task. Thus, the explanation might fail to support users in their trust calibration process due to factors such as avoidance or refusal.

**Figure 7:**
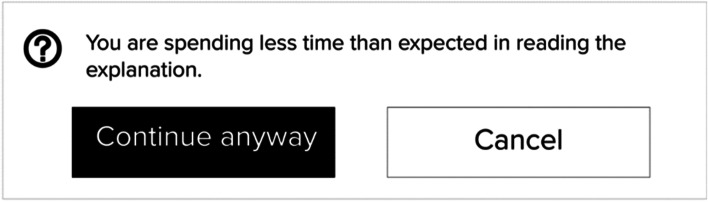
Friction design example for calibrated trust goal

This finding shifts from usable design to friction design for calibrated trust when designers for explainable interfaces might adopt techniques to combat explanation avoidance. Friction design is user experience defined as interactions that hinder people from painlessly achieving their goals when interacting with technology [46]. For instance, explanation interface designers might use anticipating possible errors technique which considers user performance as a metric and warns whether an action might cause a problem, e.g., a user spent a short time reading the explanation (See Figure 7). Another technique could be increasing the steps of making the final decision where the explanation is presented during multiple steps. The active obstruction and forced delay of the task completion caused by such techniques are likely to generate an enhanced understanding by slowing down the speed of users’ actions. These techniques have shown an effective way to increase users’ level of understanding and enable consciousness when interacting with the presented information [19, 46]. Overall, slowing users down can facilitate a reflection on their actions and this might be crucial for effective calibrated trust explanation utilisation. However, the obtrusiveness of such techniques can lead to reactance [14], i.e., the user feels that their freedom of choice has been taken away from them and they become more inclined to reject the XAI altogether.

### Support training and learning

During the Exploration phase, some participants failed to apply the explanation in their decision-making task due to reasons related to lack of familiarity and knowledge. They misinterpreted the explanation, mistrust the provided explanation or looked for confirmatory information. This finding relates to earlier work in Human-AI trust calibration [18], which showed that training clinicians to use the AI-based decision-making tool reduced users’ errors and increased the Human-AI collaborative decision-making performance. The training included learning optimal ways of using the AI in their settings and pointing out possible errors [18]. Thus, relative to Human-AI interaction research, human-explanation calibrated trust design may need to facilitate correct explanation interpretation and learn optimal usage scenarios before using XAI interface.

Furthermore, we observed that several participants’ designs considered the XAI interface as a learning interface, where users can learn from the explanations. Participants used several interactive techniques, such as tuning and grouping, to generate several instances and observations from the explanations and compare them together. This helped them to extract new knowledge from the explanation and improve their mental model. Participants’ designs tended to encourage users to look for alternative options to learn from them. For instance, P19 designed the Confidence explanation as a way of drawing her attention to missing actions, which the AI could provide it to them through a large amount of processing data and processing power. Specifically, she wanted to know how the AI thinks its certainty can achieve higher values, e.g., asking the doctor to request a further blood test to increase the AI decision accuracy. Some participants also designed explanation interfaces with hyperlinks to medical databases and research references. Their expectations of the XAI interface can be strongly anchored to their prior experience; this can sometimes require the XAI interface to provide a systematic and argumentative discussion.

We also recommend future work to apply principles of self-learning to facilitate users’ leaning process from the XAI interface [34]. As such, self-learning can improve users’ learning from the XAI interface and refresh their knowledge about AI for the most effective way to calibrate trust. It would be likely to increase the chance that they attain the desirable trust calibration behaviour over time. For instance, allowing users to write notes about the explanation, link them together, archive explanations for future comparisons and share their explainability experience with other system users.

## Conclusion

The dynamic nature of AI-based decision-making tools poses new requirements for developing interfaces with a calibrated trust goal in mind, specifically when explanations are presented. In this paper, we have conducted a qualitative approach that provides a detailed look at explainability and trust calibration. Our approach consisted of two qualitative phases: (a) exploration phase, which aims to provide a contextual understanding of the problem; (b) design phase to reveal main concepts and designs techniques that improve the role of explanation in trust calibration. Our work presents a broad view of Human-AI collaborative decision-making tools and raises important questions for future work. In particular, our design implications point towards supporting the interface design with techniques and principles to increase users’ interaction with the XAI interface to help trust calibration. For example, our results suggest that presenting explanations for trust calibration should mainly be designed to avoid undesired behaviour such as skipping explanation and habits formation. This is in-line with what we have proposed although at this stage of our research we are unable to pair between the configurations of the XAI model and the type of bias and error. Future work shall focus on the balance between making explanation effective enough in trust calibration and, simultaneously, avoiding the potential harm to user experience and being seen as a persuasive tool instead of critical thinking aid. Such neutrality in the recommendation as well as ensuring a reflective and measured reasoning in the users can be hard to achieve, especially that other requirements like engagement and design for friction are also in place.

## Footnotes

1 https://freehand.invisionapp.com/freehand/new.
